# Ultra-High Vacuum Cells Realized by Miniature Ion Pump Using High-Efficiency Plasma Source

**DOI:** 10.3390/s24124000

**Published:** 2024-06-20

**Authors:** Yuichi Kurashima, Atsuhiko Maeda, Naoto Oshima, Taisei Motomura, Takashi Matsumae, Mitsuhiro Watanabe, Hideki Takagi

**Affiliations:** 1 Device Technology Research Institute, National Institute of Advanced Industrial Science and Technology, Tsukuba 305-8564, Japan; 2 College of Science and Technology, Nihon University, Funabashi 274-8501, Japan; 3 Sensing System Research Center, National Institute of Advanced Industrial Science and Technology, Tosu 841-0052, Japan

**Keywords:** ultra-high vacuum cell, miniature ion pump, cold atom, penning vacuum gauge

## Abstract

In recent years, there has been significant interest in quantum technology, characterized by the emergence of quantum computers boasting immense processing power, ultra-sensitive quantum sensors, and ultra-precise atomic clocks. Miniaturization of quantum devices using cold atoms necessitates the employment of an ultra-high vacuum miniature cell with a pressure of approximately 10^−6^ Pa or even lower. In this study, we developed an ultra-high vacuum cell realized by a miniature ion pump using a high-efficiency plasma source. Initially, an unsealed miniature ion pump was introduced into a vacuum chamber, after which the ion pump’s discharge current, depending on vacuum pressures, was evaluated. Subsequently, a miniature vacuum cell was fabricated by hermetically sealing the miniature vacuum pump. The cell was successfully evacuated by a miniature ion pump down to an ultra-high vacuum region, which was derived by the measured discharge current. Our findings demonstrate the feasibility of achieving an ultra-high vacuum cell necessary for the operation of miniature quantum devices.

## 1. Introduction

Quantum technology exemplified by quantum computers possessing formidable processing capabilities, ultra-sensitive quantum sensors, and ultra-precise atomic clocks has garnered considerable attention in recent years. An atomic clock, known as NMIJ-F2, has been previously developed utilizing cesium (Cs) cold atoms and serves as the primary frequency standard at the National Metrology Institute of Japan [1,2,3,4]. However, applications of this atomic clock are limited to only the primary frequency standard because of its huge size. Hence, if the total system can be miniaturized to a desktop size, quantum phenomena using cold atoms could be widely applicable to other quantum devices mentioned above [5]. To achieve such a cold atomic state for those quantum devices, an ultra-high vacuum miniature cell with a pressure of approximately 10^−6^ Pa or even lower is required.

Various micro-devices can be hermetically sealed to ensure device performance, and many hermetic sealing technologies have been developed to date [6,7,8]. Even when the cell is hermetically sealed within an ultra-high vacuum environment, maintaining the hermetically sealed cell (cold atom generation chamber) at an ultra-high vacuum pressure level presents a challenge. This difficulty arises due to gas generation from the inner surfaces of the cell or gas permeation from the exterior through the bonding interface and cell walls. Moreover, achieving hermetic sealing under ultra-high vacuum conditions is technically challenging in itself. The hermetically sealed cell must be actively evacuated to maintain the cell at an ultra-high vacuum pressure level. Therefore, the miniaturization of an ultra-high vacuum cell and a vacuum pump, as well as their integration, constitutes a critical technology for miniature quantum devices.

Generally, to achieve an ultra-high vacuum environment, the vacuum chamber needs to be evacuated using high-vacuum pumps, such as the turbo molecular pump, getter pump, ion pump, cryopump, and others. Among these ultra-high vacuum pumps, the ion pump emerges as a strong candidate for realizing an ultra-high vacuum cell due to its adaptability to the MEMS (microelectromechanical systems) process. T. Grzebyk et al. have proposed the fabrication of miniature ion pumps through the process of anodic bonding of Si and glass. Additionally, they have successfully realized an ultra-high vacuum in a miniature cell [9,10,11,12].

The ion pump consists of an anode flanked by two cathodes on either side. Magnets are placed outside the anodes to apply a magnetic field to the space between the electrodes. When a high voltage is applied across the electrodes to generate plasma, the ions within the plasma collide with the titanium (Ti) layer coated on the cathode, resulting in the sputtering of Ti atoms. Sputtered Ti atoms adhere to the inner surface of the pump, forming a getter film that adsorbs active gases. Some of the ions directed toward the cathode penetrate into the cathode. In this way, not only active gases but also inert gases can be evacuated. Detailed explanations of this process have been previously published elsewhere [13]. However, when trying to reduce the size of the ion pump, the reduction in the distance between the electrodes leads to a shortened electron flight distance. This reduction makes it difficult to generate the discharge and diminishes the vacuum pumping capability. To address this issue, we have previously proposed a highly efficient miniature plasma source utilizing a magnetic mirror trap, which employs two opposing permanent magnets [14].

This study presents the successful realization of ultra-high vacuum cells through the utilization of a miniature ion pump integrated with a high-efficiency plasma source, which used the new-type magnetic circuit we have reported in [14]. The geometry of the ion pump is employed not only in pumps but also in Penning vacuum gauges. Consequently, vacuum pressure can be deduced from the discharge current of the ion pump. To measure the discharge current of the ion pump as a function of vacuum pressures, an unsealed miniature ion pump was fabricated and subsequently installed inside a vacuum chamber. The vacuum pressure inside the pump was regulated by changing the pressure of the vacuum chamber. Subsequently, a vacuum cell equipped with the miniature vacuum pump was fabricated, and a hermetically sealed space (a component of the cell) was evacuated using the integrated ion pump. The evacuation characteristics were evaluated from the discharge current of the ion pump integrated into the hermetically sealed cell. In this study, the cell was successfully evacuated to ultra-high vacuum levels, achieving a pressure of 10^−6^ Pa and underscoring the feasibility of employing miniature ion pumps for the operation of quantum devices.

## 2. Experimental

**Figure 1:**
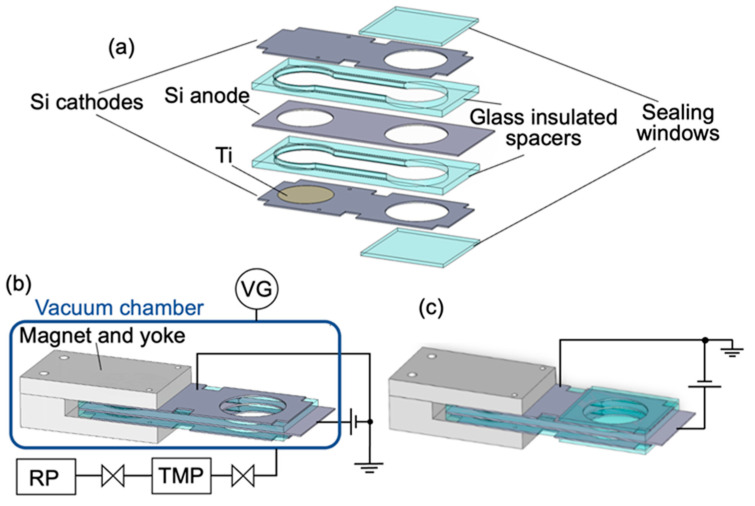
(**a**) Structure of the ion pump and the integrated cell. (**b**) The arrangement and electrical wiring diagram for evaluating the discharge characteristics of the ion pump based on the vacuum pressure. (VG: Vacuum Gauge, RP: Rotary Pump, TMP: Turbo-Molecular Pump). (**c**) Arrangement and electrical wiring diagram for evaluating the evacuation characteristics of the ion pump integrated within the sealed cell.

Figure 1a shows a breakdown diagram for each component of the miniature ion pump and the hermetically sealed vacuum cell. A silicon electrode with dimensions of 65 mm × 24 mm × 0.4 mm was employed as the anode. The silicon electrode featured two holes with diameters of 16 mm and 18 mm. Two silicon electrodes with dimensions of 60 mm × 24 mm × 0.4 mm were also placed on both sides of the anode. The cathode featured a hole with a diameter of 18 mm and six sections, which were cut off in squares to facilitate electrical contact during the anodic bonding process. The inner surfaces of the silicon cathodes were covered with thin Ti layers with a thickness of 1 μm. The silicon electrodes were also electrically insulated using a borosilicate glass with dimensions of 60 mm × 24 mm × 2 mm. The borosilicate glass featured two holes, with diameters of 17 mm and 18 mm, respectively, interconnected as depicted in Figure 1a. This configuration allowed for the monitoring of vacuum pressure within the sealed cell via the discharge current. The previously mentioned five layers, consisting of the silicon electrodes and the glass plates, were anodically bonded to each other. After degassing at a pressure of 10^−4^ Pa, the anodic bonding was conducted in the bonding chamber filled with Ne gas (>99.999%) at a pressure of 1 Pa, which is easy for generating a discharge.

Figure 1b illustrates the arrangement and electrical wiring diagram for evaluating the discharge of the ion pump integrated within the unsealed structure. We call this structure combined with the magnetic circuit an “unsealed ion pump”. To measure the discharge current of the ion pump as a function of vacuum pressure, the unsealed ion pump was installed within the small vacuum chamber made of borosilicate glass with a volume of about 360 cm^3^. To achieve a high-efficiency miniature plasma source utilizing a magnetic mirror trap, magnets embedded within a yoke were mounted outside the electrode section of the ion pump, according to our previous work [14]. After setting up the unsealed ion pump, the small vacuum chamber was evacuated using a turbo molecular pump and dry scroll vacuum pump down to an ultra-high vacuum. Here, to achieve an ultra-high vacuum, the entire vacuum chamber was annealed at 110 °C for at least 24 h using a ribbon heater for degassing. The pressure in the vacuum chamber was measured by an ionization vacuum gauge. Voltage was applied between the electrodes of the ion pump using a high-power source meter unit (SMU) (Keithley 2657A, Cleveland, OH, USA). This SMU is capable of precisely measuring currents down to a minimum of 1 fA while applying high voltage.

Figure 1c depicts the arrangement and electrical wiring diagram for evaluating the pumping characteristics of the ion pump integrated within the sealed cell. Here, two sealing windows, each with a thickness of 1 mm, were bonded to both sides of the Si cathode under a vacuum pressure of 1 Pa. In this pressure region, discharge was easily generated. Magnets embedded within a yoke were also mounted outside the electrode section of the ion pump.

## 3. Results and Discussion

**Figure 2:**
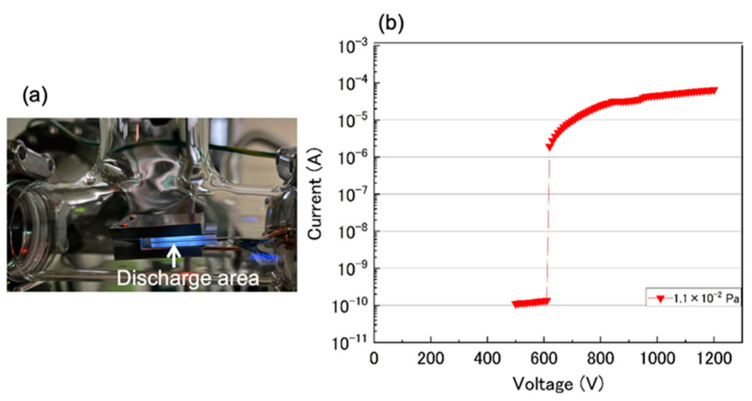
(**a**) Photograph of the glow discharge in the integrated ion pump section at low vacuum pressure. (**b**) Discharge current based on the applied voltage at a vacuum pressure of 1.1 × 10^−2^ Pa.

Figure 2a presents a photograph of the glow discharge occurring under the pressure of several Pa at 400 V. This image confirms that a glow discharge could be successfully generated at the brightly illuminated section inside the integrated ion pump. Figure 2b shows the discharge current depending on the applied voltage at a lower vacuum pressure of 1.1 × 10^−2^ Pa. The applied voltage was gradually increased from 500 to 1200 V in increments of 10 V to examine the discharge initiation. The current experienced a sudden increase when a voltage of approximately 600 V was applied. The discharge current gradually increased as the increasing voltage was raised up to 1200 V.

**Figure 3:**
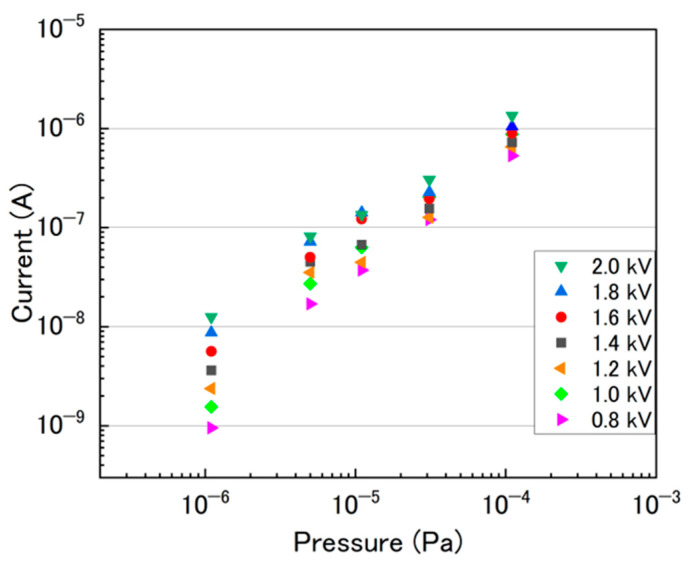
Discharge currents measured as a function of vacuum pressure for each applied voltage.

Figure 3 displays the measured discharge currents of the unsealed ion pump as a function of vacuum pressure. The discharge current of the unsealed ion pump was measured at applied voltages ranging from 800 to 2000 V, under vacuum pressures in the order of 10^−4^ Pa to 10^−6^ Pa. In the ultra-high vacuum region, it took several seconds before a discharge generation occurred, even when a high voltage was applied. We confirmed that the current value before discharge generation was lower in more than two orders of magnitude, as shown in Figure 2b. One should note that generating plasma in high vacuum conditions is generally challenging. For this reason, 2000 V (the highest voltage) was initially applied to the ion pump, and it was subsequently reduced from 2000 to 800 V in increments of 200 V each. At a discharge voltage of 2000 V, the discharge current was 1.4 × 10^−6^ A at 1.1 × 10^−4^ Pa, 1.3 × 10^−7^ A at 1.1 × 10^−5^ Pa, and 1.3 × 10^−8^ A at 1.1 × 10^−6^ Pa. It was found that the discharge current decreased almost proportionally to the decrease in vacuum pressure across all applied voltages. For a given vacuum pressure, the magnitude of the discharge current tended to decrease with a reduction in the applied voltage. This trend was especially pronounced at the lowest pressure of 1.1 × 10^−6^ Pa. This occurs because, as the degree of vacuum decreases, the ionization of residual gas molecules diminishes due to a reduction in the number of residual gas molecules.

**Figure 4:**
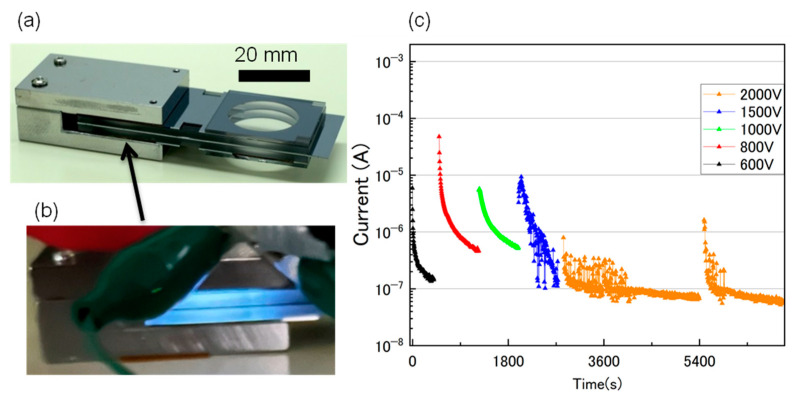
(**a**) Photograph of the vacuum cell achieved by integrating an ion pump into a sealed cell. (**b**) Photograph of a glow discharge emanated from the ion pump section immediately after hermetic sealing at a vacuum pressure of 1 Pa. (**c**) Discharge current as a function of discharge time across applied voltages.

Figure 4a presents a photograph of the vacuum cell achieved by integrating the ion pump into a sealed cell. Magnets embedded into a yoke were also mounted outside the electrode section of the ion pump. Figure 4b displays a photograph of the glow discharge emanating from the ion pump section immediately after hermetic sealing, at a vacuum pressure of 1 Pa. Even though this cell was sealed in a vacuum at a pressure of 1 Pa, it can be inferred that the vacuum pressure inside the cell was in the order of several tens of Pa, based on the ignition voltage at 380 V. It should be noted that the vacuum level slightly deteriorated due to degassing after sealing. Therefore, the pressure value of 10 Pa inside the cell appears to be a reasonable estimate. Figure 4c illustrates the variations in the discharge current alongside the voltage application time. Constant voltages were applied between the electrodes, and the discharge currents were measured every 5 s as a function of time. Here, the applied voltage was increased from 600 V to a maximum of 2000 V. Relatively large currents were measured just after voltage application, indicating pressure increase inside the cell without pump operation. The discharge current drastically decreased immediately after applying the voltage, indicating pumping operation. After a period, the rates of diminution in the discharge current gradually decreased for each voltage. The discharge current exhibited instability when 1500 V was applied. Moreover, when 2000 V was applied, the current initially showed instability, but subsequently stabilized and began to decrease gradually. The reason for this instability of current is not clear at this time. We believe that further investigation is needed to reveal the behavior.

**Figure 5:**
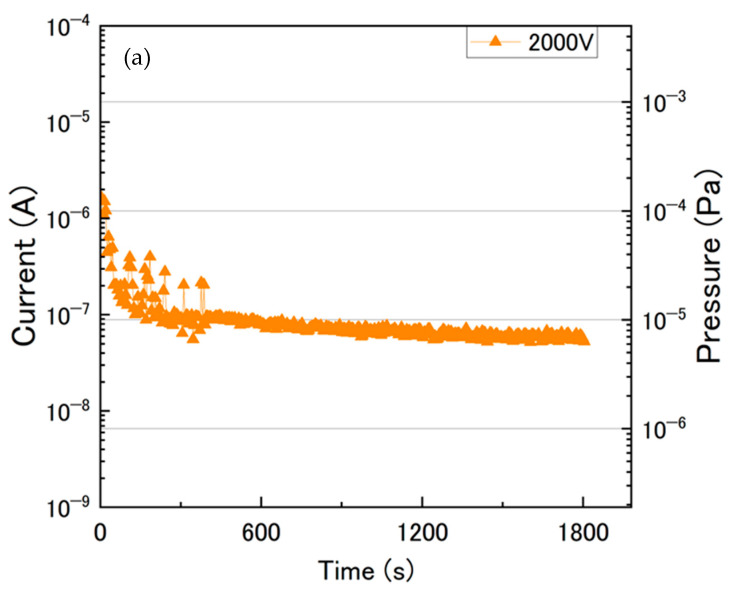

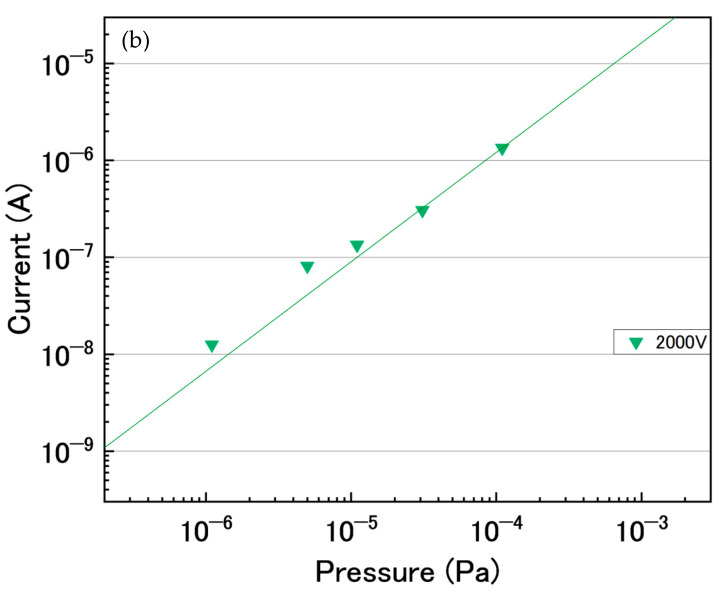
(**a**) Discharge current and vacuum pressure as a function of discharge time with an applied voltage of 2000 V. Left vertical axis shows the discharge current. Right vertical axis represents the vacuum pressure derived using the fitting data in (**b**). (**b**) The fitting line of the discharge current as a function of the vacuum pressure at an applied voltage of 2000 V in the graph in Figure 3.

Figure 5a shows the discharge current as a function of discharge time at 2000 V. Vacuum pressure inside the cell estimated from the discharge current is also shown on the left side. Here, the discharge current I generated by a cold cathode discharge has the following relationship to the pressure P below a vacuum level of 10^−2^ Pa. The real dependence of I on P is given by:

(1) $$I=K{\cdot P}^{n}$$

where K and n depend on the pump parameters, including the gas species, electrode structure, magnetic field, applied voltage, etc. The value of the constant *n* is typically between 1 and 1.2 [15]. By fitting approximate Formula (1) to the data acquired for 2000 V, as shown in Figure 3, the conversion Equation (2) for the degree of vacuum with respect to the discharge current can be obtained:

(2) $$I=0.040\pm 0.005{\cdot P}^{1.130\pm 0.017}$$

Figure 5b shows the fitting line of Equation (2).

The pressure values on the right vertical axis in Figure 5a are derived from this formula. Finally, the estimated pressure was 6 × 10^−6^ Pa. We conclude that the cell can be evacuated by the developed miniature ion pump down to the ultra-high vacuum region, which was derived from the discharge current.

## 4. Conclusions

This study proposed an ultra-high vacuum cell integrated with a miniature ion pump featuring a high-efficiency plasma source. First, the discharge current of the ion pump was evaluated depending on vacuum pressure using an unsealed structure. Subsequently, we achieved the integration of the ultra-high vacuum cell with the miniature ion pump through hermetic bonding, and the evacuation characteristics were assessed based on the discharge current of the integrated ion pump. This study’s findings imply that the developed miniature ion pump is capable of evacuating the cell to ultra-high vacuum levels.

## Data Availability

The data presented in this study are available upon request from the corresponding author.
